# Clinical Prognostic Factors and Integrated Multi-Omics Studies Identify Potential Novel Therapeutic Targets for Pediatric Desmoid Tumor

**DOI:** 10.1186/s12575-022-00180-0

**Published:** 2022-12-20

**Authors:** Bo Ning, Peng Huang, Lining Zhu, Zhijie Ma, Xiaoli Chen, Haojun Xu, Ruixue Ma, Chengyun Yao, Pengfei Zheng, Tian Xia, Hongping Xia

**Affiliations:** 1 Department of Paediatric Orthopedics, Children’s Hospital of Fudan University, Shanghai, 201102 China; 2 Department of Paediatric Orthopedics, Hainan Women and Children’s Medical Center, Haikou, 570206 China; 3 Department of Pathology, Nanjing Drum Tower Hospital & Drum Tower Clinical College & Key Laboratory of Antibody Technique of National Health Commission, Nanjing Medical University, Nanjing, 211166 China; 4 Sir Run Run Hospital, Nanjing Medical University, Nanjing, 211166 China; 5 Jiangsu Cancer Hospital & The Affiliated Cancer Hospital of Nanjing Medical University& Jiangsu Institute of Cancer Research, Nanjing, 2100092 China; 6 Department of Orthopedics Surgery, Children’s Hospital of Nanjing Medical University, Nanjing, 210008 China

**Keywords:** Clinical prognostic factors, Whole exome sequencing, RNA sequencing, Untargeted metabolomics profiling, Pediatric desmoid tumor

## Abstract

**Background:**

Desmoid tumor (DT), also known as desmoid-type fibromatosis (DTF) or aggressive fibromatosis (AF) is a rare mesenchymal tumor affecting both children and adults. It is non-metastasis but infiltrative, growing with a high recurrence rate to even cause serious health problems. This study investigates the biology of desmoid tumors through integrated multi-omics studies.

**Methods:**

We systematically investigated the clinical data of 98 extra-abdominal cases in our pediatric institute and identified some critical clinical prognostic factors. Moreover, our integrated multi-omics studies (Whole Exome Sequencing, RNA sequencing, and untargeted metabolomics profiling) in the paired PDT tumor/matched normal tissues identified more novel mutations, and potential prognostic markers and therapeutic targets for PDTs.

**Results:**

The top mutation genes, such as CTNNB1 (p.T41A and p.S45F) and MUC4 (p.T3775T, p.S3450S, etc.), were observed with a mutation in more than 40% of PDT patients. We also identified a panel of genes that are classed as the FDA-approved drug targets or Wnt/β-catenin signaling pathway-related genes. The integrated analysis identified pathways and key genes/metabolites that may be important for developing potential treatment of PDTs. We also successfully established six primary PDT cell lines for future studies.

**Conclusions:**

These studies may promote the development of novel drugs and therapeutic strategies for PDTs.

**Supplementary Information:**

The online version contains supplementary material available at 10.1186/s12575-022-00180-0.

## Introduction

A desmoid tumor (DT), also known as desmoid-type fibromatosis (DTF) or aggressive fibromatosis (AF), is a kind of rare mesenchymal tumor with aggressive fibroblastic proliferation in the intra- and extra-abdominal localization. It is a non-metastasizing mesenchymal neoplasm of deep soft tissue but infiltrative slow or aggressive growing and locally invasive with a high recurrence rate. It is a rare tumor with an incidence of 5–6 cases per million per year and affects both children and adults [1]. It is not life-threatening but causes problems or even death when they are aggressively growing and compress other important organs. Surgery was previously the standard primary treatment [2]. However, more conservative management has been agreed upon among clinicians in recent years. A joint global consensus-based guideline approach for the management of adult and pediatric patients with DTs has been agreed upon in a global consensus meeting held in Milan, Italy, in June 2018 by The Desmoid Tumor Working Group. This guideline focused on molecular genetics, indications for active treatment, and available systemic therapeutic options [3, 4].

We previously observed that pediatric DT (PDT) was commonly located in the buttocks and lower extremities in our pediatric institute [5]. After the diagnosis of DTs, the front-line approach is active surveillance [6]. Only in case of progression active treatment may be applied. There are many different clinical prognostic factors for pediatric extra-abdominal desmoid tumors. Previously, we have reviewed clinical data of 66 extra-abdominal pediatric desmoid tumor patients who underwent macroscopically complete resection and radiotherapy for some recurrent tumors. We found that age, sex, tumor site, tumor size, and proximity to nerves/vasculature had a significant impact on prognosis in univariate analysis, while younger age, tumor location in buttocks, larger tumor, and proximity to essential nerves/vasculature were independent risk factors for poor prognosis in multivariate analysis. Thirty-six (55%) patients had local recurrence with a median follow-up of 6.6 years. Radiotherapy decreased the local recurrence rate and should be considered [7]. Since surgery is less frequently undertaken for DTs in the latest guideline, the level of evidence for adjuvant radiotherapy is low, except for patients with difficult treatment recurrent disease. Besides surgery and radiotherapy, systemic treatment options may be applied for DTs, which mainly include tyrosine kinase inhibitors (TKIs), non-steroidal anti-inflammatory drugs (NSAIDs), “low-dose” chemotherapeutic drugs, and antihormonal therapies [3, 4].

The cause of most desmoid tumors is still unclear. It is crucial for an understanding of the biology of these rare tumors. Previous genetic studies have shown most sporadic DTs harbor alterations in CTNNB1 [8–10]. Additional integrated multi-omics studies may be needed to identify potential novel prognostic markers and therapeutic targets for pediatric desmoid tumors, which may promote the development of novel drugs and enhance therapeutic strategies. In the present study, we further reviewed clinical data of 98 extra-abdominal pediatric desmoid tumor patients who underwent macroscopically complete resection in our hospital. We performed integrated multi-omics studies based on Whole Exome Sequencing (WES), RNA sequencing (RNAseq), and untargeted metabolomics profiling for comprehensive molecular characterization of the paired pediatric DTs. Moreover, we also successfully established six primary pediatric desmoid tumor cell lines for future studies.

## Materials and Methods

### Pediatric Desmoid Tumor Specimens and Clinicopathological Features

We collected 98 PDT patients samples who underwent complete resection at the Children’s Hospital of Fudan University (CHFU, Shanghai, China) from 2004 to 2020. This study was approved by the Clinical Institutional Review Board (IRB) of our institutes. The sample collection was agreed upon with the guardian of each patient. All samples were confirmed with DT diagnosis by histopathology and molecular analysis based on the guidance. The clinicopathological features of 98 patients according to tumor presentations are shown in Table 1.

**Table 1:** Characteristic of 98 patients according to tumor presentations

Characteristic	Overall (***n*** = 98)	Primary	Recurrent	***P***-value
Total	98	32 (32.6%)	66 (67.4%)	
**Gender**				**0.062**
Male	64 (65.3%)	18 (28.1%)	46 (71.8%)	
Female	34 (34.7%)	13 (38.2%)	20 (61.8%)	
**Age (yrs)**				**0.000/0.001**
0–5	44 (44.9%)	12 (27.3%)	32 (72.7%)	
6–10	29 (29.6%)	6 (20.7%)	23 (79.3%)	
> 10	25 (25.5%)	14 (56%)	11 (44%)	
**Location**				**0.026/0.000**
Extremity	30 (30.6%)	11 (36.7%)	19 (63.3%)	
Trunk	19 (19.4%)	11 (57.9%)	8 (42.1%)	
Buttock	49 (50%)	10 (20.4%)	39 (79.6%)	
**Size (cm)**				**0.000/0.000/0.0018**
0–5	17 (17.3%)	11 (64.7%)	6 (35.3%)	
6–10	42 (42.9%)	14 (33.3%)	28 (66.7%)	
> 10	39 (39.8%)	7 (17.9%)	32 (82.1%)	
**Adjacent to nerves/vascular**				**0.000**
Yes	43 (43.9%)	8 (18.6%)	35 (81.4%)	
No	55 (56.1%)	24 (43.6%)	31 (56.4%)	

### Library Preparation and Whole Exome Sequencing (WES)

After the isolation of genomic DNA, There are two main methods of QC for DNA samples. We test DNA degradation and potential contamination using Agarose Gel Electrophoresis and quantify the DNA concentration precisely by Qubit 2.0 analysis. A total amount of 1.0 μg genomic DNA per sample was used for library preparation and WES. WES with Illumina sequencers was done by Novogene Co., Ltd. The detail was described in the Supplementary.

### Library Preparation and RNA Sequencing (RNAseq)

After the isolation of total RNA, the QC for RNA samples was examined by Nanodrop, Agarose Gel Electrophoresis and Agilent 2100. A qualified enough amount of total RNA was used for library preparation and RNA sequencing. RNAseq with Illumina sequencers was done by Novogene Co., Ltd. The detail was described in the Supplementary Materials and Methods.

### The Untargeted Metabolomics Profiling

The untargeted metabolomics profiling was performed on the XploreMET platform (Metabo-Profile, Shanghai, China). The paired frozen pediatric desmoid tumor specimens were sent to the Metabo-Profile with liquid nitrogen, and the sample preparation procedures are referred to in their protocols. The detail was described in the Supplementary Materials and Methods.

### Establishment of the Primary Pediatric Desmoid Tumor Cell Lines

Primary cell cultures from different DT tissue samples were collected during surgical resection. The DT tissues were immediately cut into small pieces and incubated with collagenase solution for 18 to 24 hours at 37 °C. Then the cell suspension was filtrated through 100 μm filters. The dissociated cells were seeded into different culture dishes. The cells were passaged after the cells near confluence and grown in DMEM with 15% FBS. The established primary culture cells were examined to ensure that the cells were representative of the primary pediatric desmoid tumor cells by performing immunohistochemistry (IHC) staining and sequencing analysis.

### Bioinformatics and Statistical Analysis

Raw sequencing output from WES and RNAseq was transferred from the sequencing instrument to a bioinformatics server for professional bioinformatics analysis. Briefly, WES data were aligned to the human reference genome build hg38 with bwa (version 0.7.15), followed by the GDC DNA-Seq analysis pipeline available from (https://docs.gdc.cancer.gov/). Somatic single nucleotide variants (SNVs) were identified using Varscan (version 2.3.9) and subjected to annotation via annovar. These SNVs were further filtered for missense and nonsense mutation and subjected to visualization via R (version 4.0.2) package maftools. The differential metabolites were obtained using univariate statistical analysis (student T-test or Mann-Whitney U test, depending on the normality of data and homogeneity of variance), especially when the multivariate OPLS-DA model fails to build a reliable discriminant model under some conditions. Pathway analysis for the significantly overexpressed genes was done by QIAGEN Ingenuity Pathway Analysis (QIAGEN IPA). Pathway enrichment analysis uses the Pathway-associated metabolite sets (SMPDB). MetaboAnalyst 5.0 Joint Pathway Analysis and Network Explorer were applied to perform integrated analysis on results obtained from combined metabolomics and gene expression studies [11]. The statistical analysis software, Partek® Genomics Suite® and IBM SPSS Statistics, were used for further statistical analysis.

## Results

### PDT Is Highly Recurrent and Critical Clinical Prognostic Factors Affect the Prognosis

Given the high recurrence of PDTs, we concluded the clinical factors of 98 cases, including age at presentation, gender, tumor size, tumor location, and relation with important vessels or nerves. To understand the clinical prognostic factors, we analyzed the associations between these clinical data and tumor recurrence. Table 1 shows the detailed clinical data of all patients. 32 of 98 cases are primary cases (32.6%) and 66 are recurrent cases (67.4%); of which 64 are boys and 34 are girls. In gender, there was no obviously statistically significance for the recurrence rate (*p* = 0.062). Kaplan-Meier method for RFS showed that male patients were of higher recurrent possibility. For the age at tumor presentation, we divided three groups with 0 to 5 years old (44, 44.9%), 6 to 10 (29, 29.6%), and over 10 years old (25, 25.5%). There was a familiar recurrence rate between two lower age groups and were of higher recurrence rates than the over 10 years of age group. RFS analysis for different age groups showed that lower age tended to tumor local recurrence. According to tumor location, we divided three groups: Buttock, Extremity and Trunk. The most frequent location is the buttock (50%) and has the highest recurrence rate (79.6%, *P* = 0.000). Tumor at the trunk has the lowest recurrence rate (42.1%). RFS analysis for tumor location showed the same results that the tumor at the buttock has the highest local recurrence tendency. Tumor size is a very important for tumor prognosis. The larger size of the tumor, the higher the recurrence rate observed (*P* = 0.000/0.002). RFS showed that the largest size group had the highest recurrence tendency. We divided them into two groups according to the connection of the tumor distance to the nerves or vessels. We found that tumors close to the nerves or vessels had a higher recurrence rate than the rest patients (*P* = 0.000). Moreover, cox regression analysis was used to evaluate clinical prognostic factors with univariate and multivariate analyses (Table 2). The independent prognostic factors included gender, age, tumor size and adjacent to vital nerves or vessels.

**Table 2:** Univariate and multivariate analysis of the effect of covariates of RFS for 66 patients

Factors	Univariate		Multivariate	
	HR (95% CI)	***P-***value	HR (95% CI)	***P-***value
Gender (male vs.female)	1.83 (1.15–2.22)	0.000	3.24 (2.56–4.09)	0.000
Age (vs.0–5 yrs)				
6–10 yrs.	2.70 (2.06–3.54)	0.000	10.53 (7.00–15.84)	0.000
> 10 yrs.	1.78 (1.39–2.27)	0.000	3.24 (2.43–4.31)	0.000
Size (vs.0-8 cm)				
9–14 cm	0.19 (0.13–0.26)	0.000	0.22 (0.14–0.32)	0.000
> 14 cm	1.71 (1.40–2.10)	0.000	1.29 (1.01–1.65)	0.043
Adjacent to nerves/vascular				
(Yes vs. No)	4.60 (3.85–5.48)	0.000	3.03 (2.34–3.84)	0.000
Location (vs. Buttock)				
Extremity	27.80 (16.32–47.37)	0.000	5.78 (3.23–10.42)	0.000
Trunk	21.71 (12.40–38.01)	0.000	8.54 (4.62–15.79)	0.000

### Whole Exome Sequencing (WES) Identified the Critical Mutations in PDTs

**Fig. 1:**
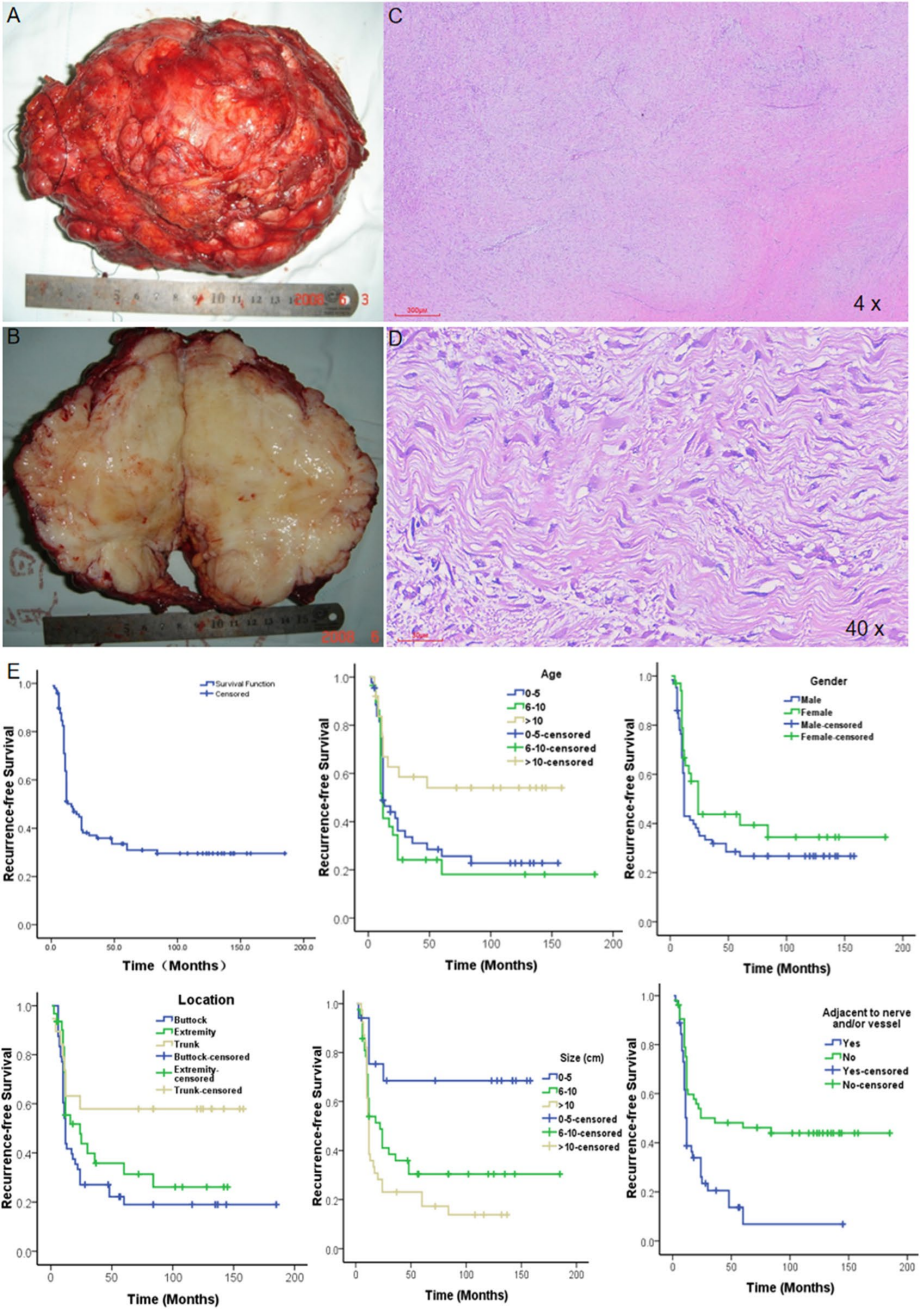
The critical clinical prognostic factors affect the prognosis of PDTs. **A**-**B** The representative gross morphology examination of complete resection PDTs. **C**-**D** The microscopic examination of tissue sections with Hematoxylin and eosin (H&E) stains under different magnifications. **E** The overall recurrence survival and various critical clinical prognostic factors (age, gender, tumor location, tumor size and adjacent to nerve and/or vessel) affect the prognosis of PDTs

**Fig. 2:**
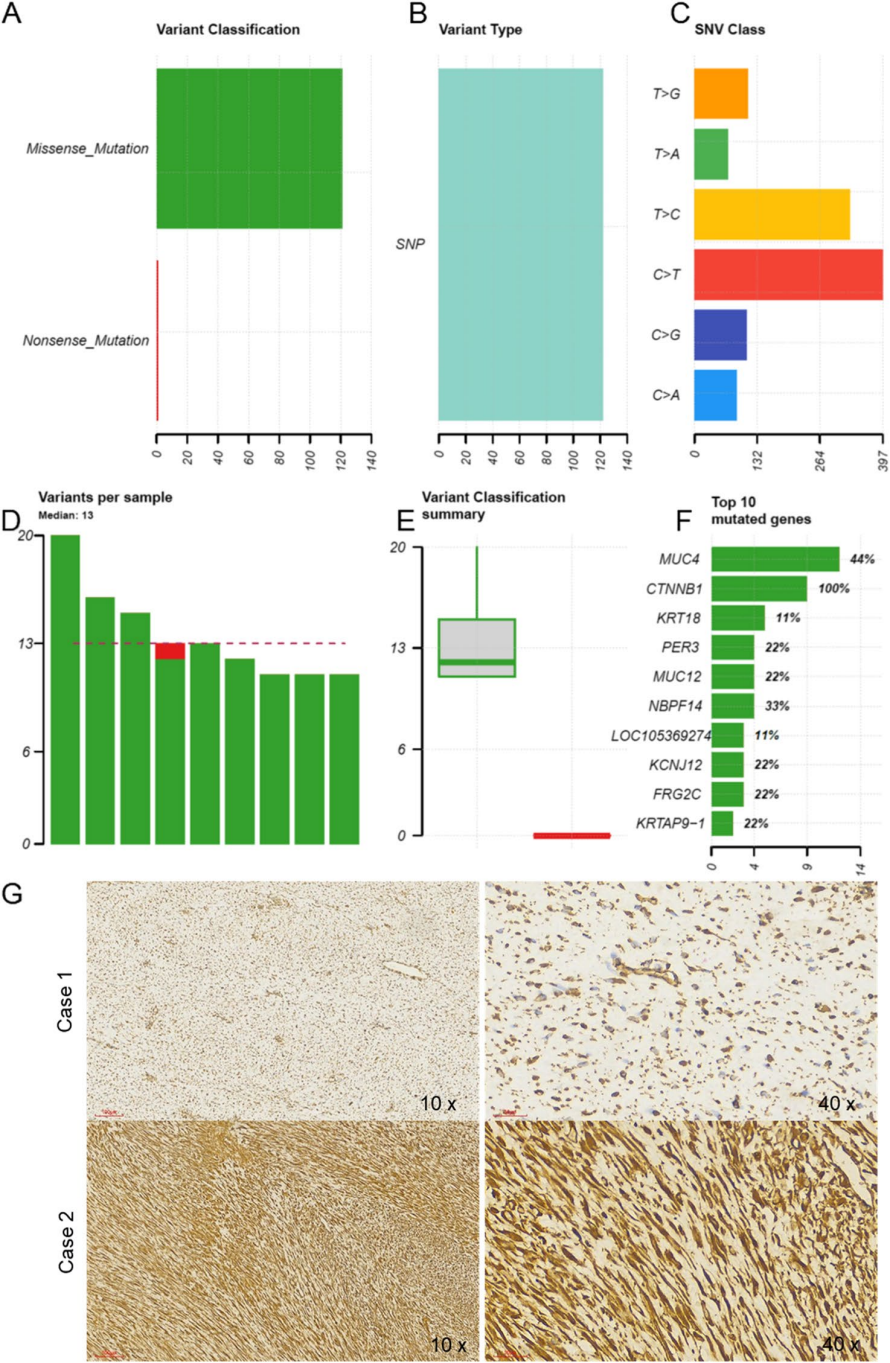
Whole Exome Sequencing (WES) identified the critical mutations in PDTs and Immunohistochemistry (IHC) analysis of β-catenin. **A** A summary of the number of mutations from indicated variant classifications in nine patients. **B** A summary of the number of mutations from indicated variant types in nine patients. **C** A summary of the number of base-transition in nine patients. **D** Comparison of the number of variants among nine patients. Red and green bars represent missense and nonsense mutation, respectively. **E** Comparison of the number of variants between indicated variant classifications across nine patients. **F** A summary of the number of variants in indicated genes. The percentage of indicated genes observed with at least one mutation in nine patients was labeled on the right of the bar. **G** The representative images of IHC staining for β-catenin in different cases with different magnifications

To understand the genomic alteration basis of pediatric desmoid tumors, we conducted WES for nine paired frozen pediatric tumor tissue samples. More than 10G raw data was sequenced for each tumor or matched normal samples. The efficiency (%) was more than 98% and the error (%) was less than 0.05% for all samples. Figure 1 shows the variant classification results. There was a median of around 13 variants per sample. The predominant mutation type was a missense single nucleotide polymorphism (SNP). In terms of the mutational spectrum, a predominance of C > T transversions was seen. The top ten most commonly mutated genes were CTNNB1 (100%, p.T41A and p.S45F), MUC4 (44%, p.T3775T, p.S3450S, etc.), NBPF14 (33%), PER3, MUC12, KCNJ12, FRG2C, KRTAP9–1(22%), KRT18, LOC105369274 (11%). All our samples were detected with CTNNB1 mutation at either chr3–41,266,137 C > T (66.67%) or chr3–41,266,124 A > G (33.33%). The IHC staining for β-catenin in different cases of PDTs with different magnifications also showed strong nuclear staining of β-catenin (Fig. 2G).

### RNA Sequencing (RNAseq) Identified the Dysregulated Genes in PDTs

**Fig. 3:**
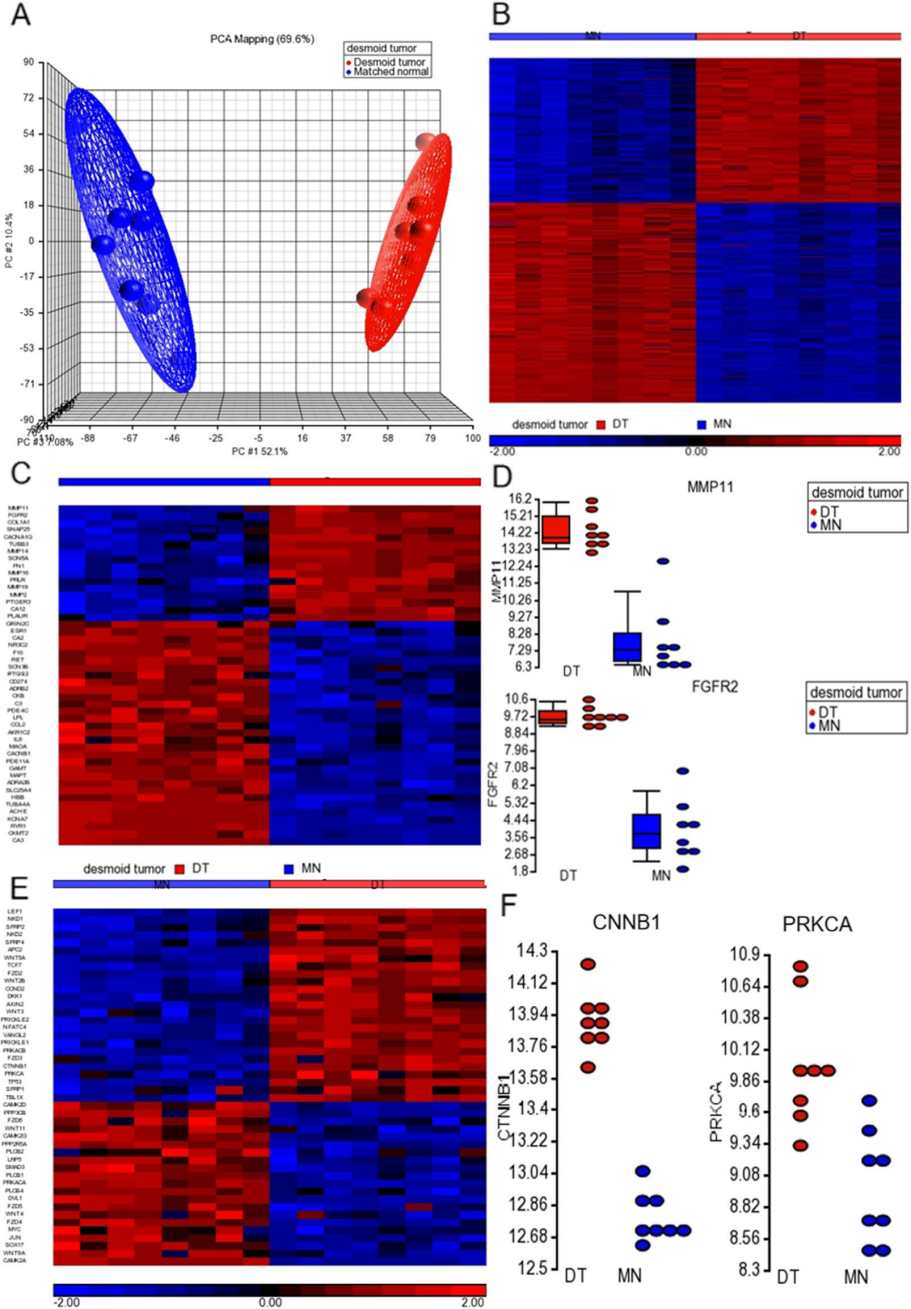
RNA sequencing (RNAseq) identified the dysregulated genes in PDTs. **A** The Principal components analysis (PCA) analysis showed the tumor was different from the matched normal samples. **B** The heat map showed around 600 genes that are significantly dysregulated in tumors compared to matched normal samples with cut-off 10 fold and P less than 0.05. **C** The heat map showed that 47 genes are classed as the FDA-approved drug targets. **D** The dot plot showed the significant expression difference between MMP11 and FGFR2 in the paired PDTs. **E** The heat map showed around 46 Wnt/β-catenin signaling pathway-related genes significantly dysregulated in PDTs. **F** The dot plot showed the significant expression difference between CTNNB1 and PRKCA in the paired PDTs

To identify the significantly dysregulated genes in desmoid tumors, we conducted the RNAseq analysis in nine paired pediatric desmoid tumor tissues. More than 6G raw data was sequenced for each tumor or matched normal samples. The effective (%) was more than 98% and the error (%) was less than 0.05% for all samples. The Principal components analysis (PCA) is a common unsupervised method for the analysis of gene expression data, which clearly showed the tumor was different from the matched normal samples (Fig. 3A). We identified around 600 genes that are significantly dysregulated in tumors compared to matched normal samples with cut-off 10 fold and P less than 0.05 (Fig. 3B). Among them, 47 genes are classed as the FDA approved drug targets, which may be potential therapeutic targets for PDTs such as MMP11 and FGFR2 (Fig. 3C and D). Since wnt/β-catenin signaling pathways are critical for PDTs, we further analyzed and observed that around 46 Wnt/β-catenin signaling pathway-related genes significantly dysregulated in PDTs, including CTNNB1 and PRKCA (Fig. 3E and F). These data suggested that a panel of dysregulated genes may contribute to the development of PDT and are potential therapeutic targets.

### The Untargeted Metabolomics Profiling Identified the Key Metabolites in PDTs

**Fig. 4:**
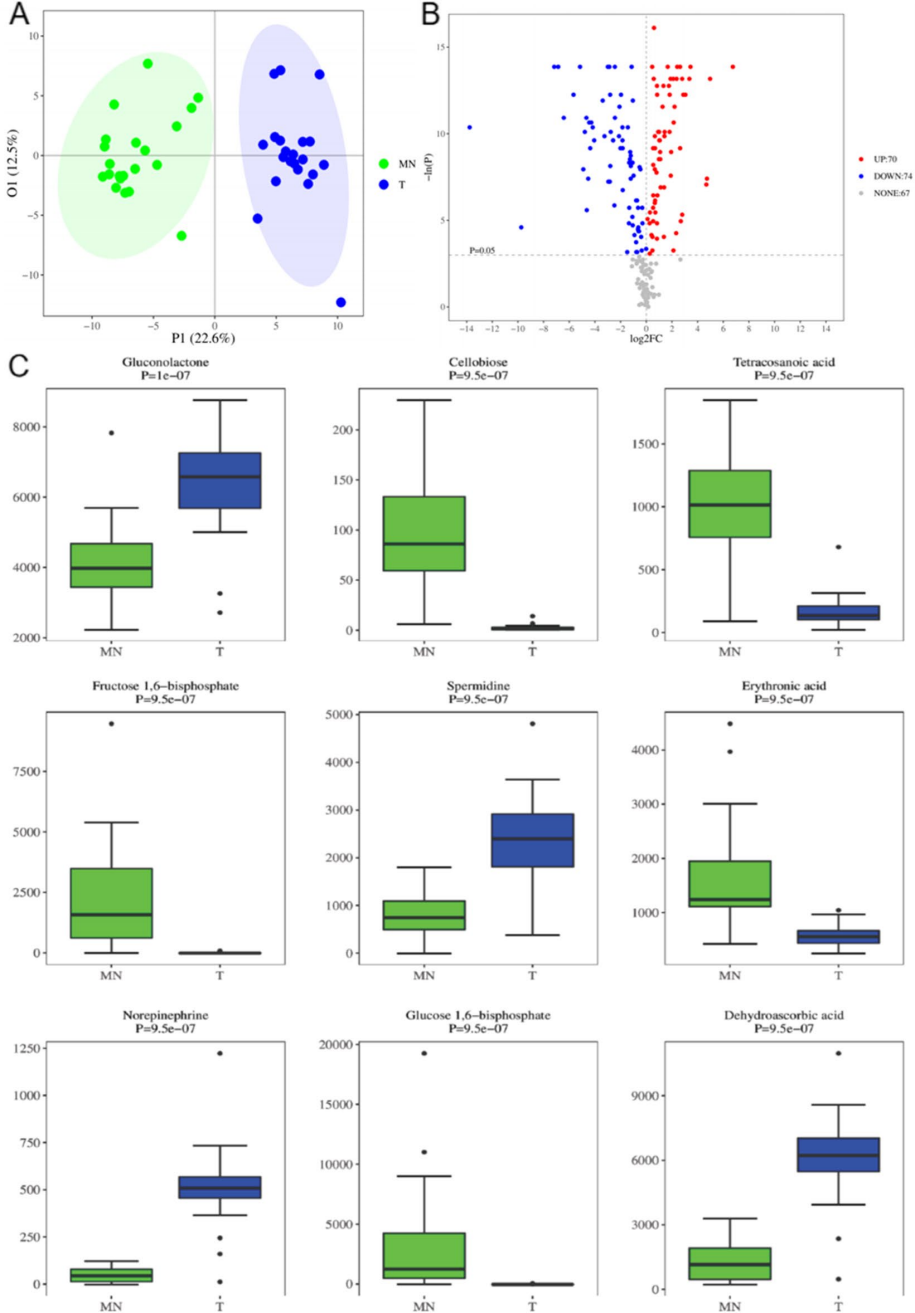
The untargeted metabolomics profiling identified the key metabolites in PDTs. **A** The OPLS-DA 2D score plot showed the distribution of the 20 paired tumor (T) /matched normal (MN) frozen tissues for the untargeted metabolomics analysis. **B** The Volcano Plot displays the fold change (FC) and *p*-value of each metabolite. The threshold value for differential metabolites selection is: (1) *P* < 0.05, (2)|log2FC| > 0. In the volcano plot, compared with MN, differential metabolites (points with red highlight) in the right top corner are increased in T and differential metabolites (points with blue highlight) in the left top corner are decreased in T. **C** The Boxplot of the top 9 differential metabolites ordered by *P*-value are shown

Next, we try to understand the metabolism dysregulation and key metabolites contributing to the PDT by the untargeted metabolomics analysis of 20 paired tumor (T) /matched normal (MN) frozen tissues. The differential metabolites were obtained using univariate statistical analysis (student T-test or Mann-Whitney U test, depending on the normality of data and homogeneity of variance), especially when the multivariate OPLS-DA model fails to build a reliable discriminant model under some conditions. The OPLS-DA 2D score plot is shown in Fig. 4A and the Volcano Plot of Univariate Statistics is shown in Fig. 4B. Volcano Plot displays fold change (FC) and *p*-value of each metabolite. In this project, threshold value for differential metabolites selection is: (1) *P* < 0.05,(2)|log2FC| > 0. In the volcano plot, compared with MN, differential metabolites (points with red highlight) in the right top corner are increased in T and differential metabolites (points with blue highlight) in the left top corner are decreased in T. The Boxplot of the top 9 differential metabolites ordered by *P*-value is shown in Fig. 4C. Threshold value for potential biomarker selection in this project is: (1) VIP > 1 in multi-dimensional statistics, (2) *P* < 0.05 and |log2FC| > 0 in univariate statistics [12, 13].

### Pathway Analysis on Results Obtained from Gene Expression and Metabolomics Studies

**Fig. 5:**
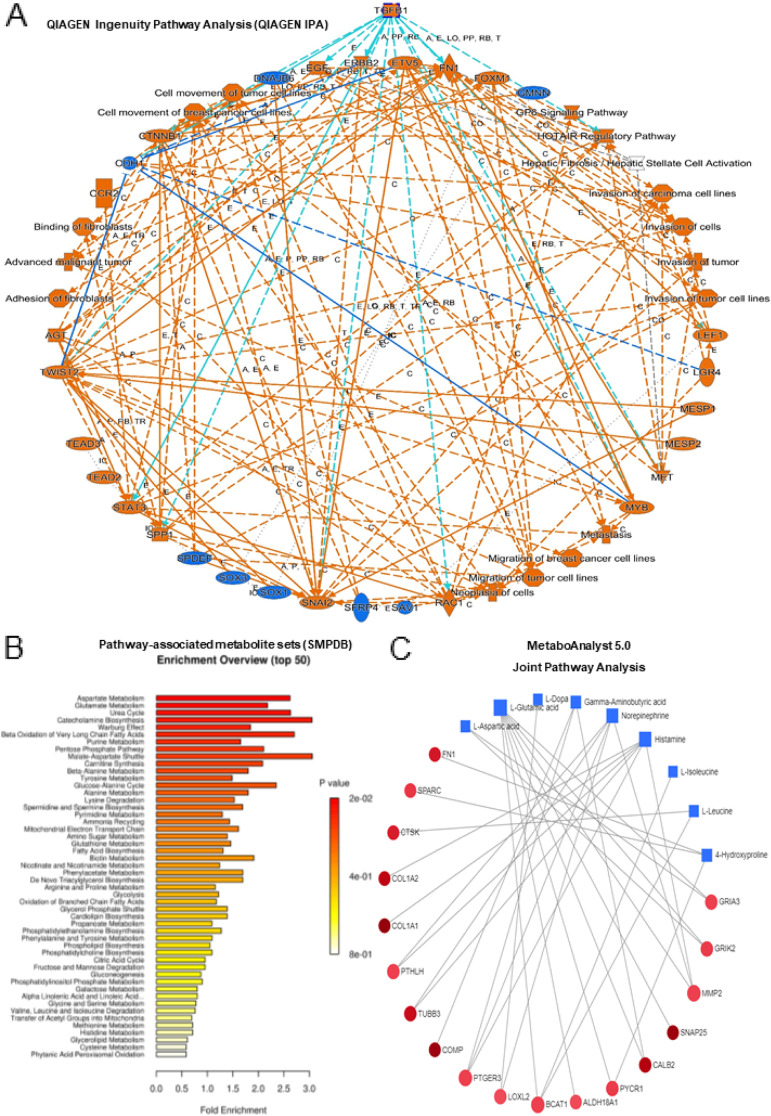
Pathway Analysis on results obtained from gene expression and metabolomics studies. **A** Graphical summary of the key pathways using the significantly overexpressed genes in PDT tumors compared to matched normal samples (cut-off 10 fold and P less than 0.05) by QIAGEN Ingenuity Pathway Analysis (QIAGEN IPA). **B** Pathway enrichment analysis using Pathway-associated metabolite sets (SMPDB) is shown for the top 50 enriched pathways. **C** MetaboAnalyst 5.0 Joint Pathway Analysis for the overexpressed genes and metabolites

**Fig. 6:**
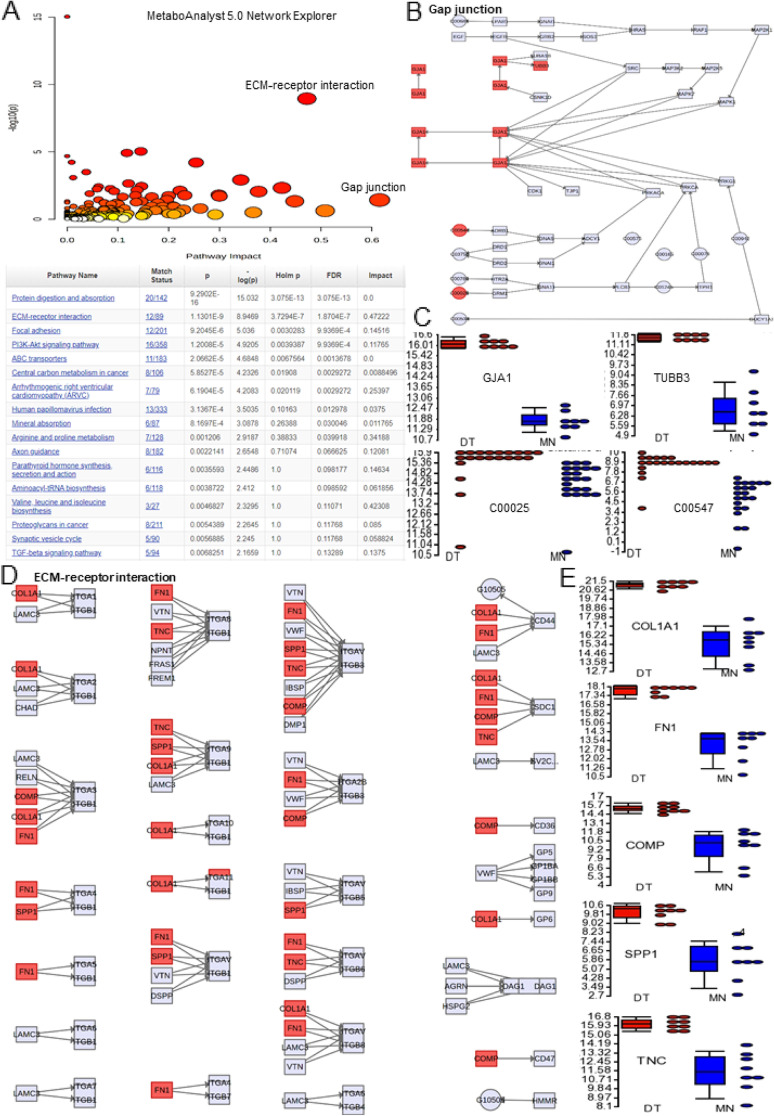
MetaboAnalyst 5.0 Network Explorer visually explores the relationships in different biological networks of the overexpressed genes and metabolites. **A** The overall pathways are involved in different biological networks of the overexpressed genes and metabolites. **B**-**C** The key overexpressed genes/metabolites involved in the Gap junction. **D**-**E** The key overexpressed genes/metabolites involved in the ECM-receptor interaction

To understand the key pathways involved the dysregulated genes and metabolites, we have investigated different pathway analyses. Figure 5A shows the graphical summary of the key pathways using the significantly overexpressed genes in PDT tumors compared to matched normal samples (cut-off 10 fold and P less than 0.05) by QIAGEN Ingenuity Pathway Analysis (QIAGEN IPA). Interestingly, we observed that TGFB1 is the key upstream regulator and control many overexpressed genes which are involved in different invasion and migration process. Pathway enrichment analysis using Pathway-associated metabolite sets (SMPDB) was also shown for the top 50 enriched pathways (Fig. 5B). Furthermore, MetaboAnalyst 5.0 Joint Pathway Analysis performs integrated pathway and connection analysis on results obtained from combined metabolomics and gene expression studies (Fig. 5C). Besides, MetaboAnalyst 5.0 Network Explorer also visually explores the relationships in different biological networks of the overexpressed genes and metabolites. Figure 6  shows the overall pathways involved in various biological networks of the overexpressed genes and metabolites. For example, the key overexpressed genes/metabolites involved in the Gap junction and ECM-receptor interaction. Gap junctions have been shown to help tumor cell invasion, and metastasis, increase nutrient supply and interact with immune cells to escape detection [14]. This may be related to the highly aggressive growth, invasion and recurrence of PDTs. The extracellular matrix has also been shown to be important in soft tissue sarcomas. Interactions between ECM ligands and their corresponding adhesion receptors are critical in driving many oncogenic processes like proliferation, invasion, altered metabolism and tumor immune microenvironment [15, 16]. These pathways and key genes/metabolites may be important for developing potential treatment of PDTs.

**Fig. 7:**
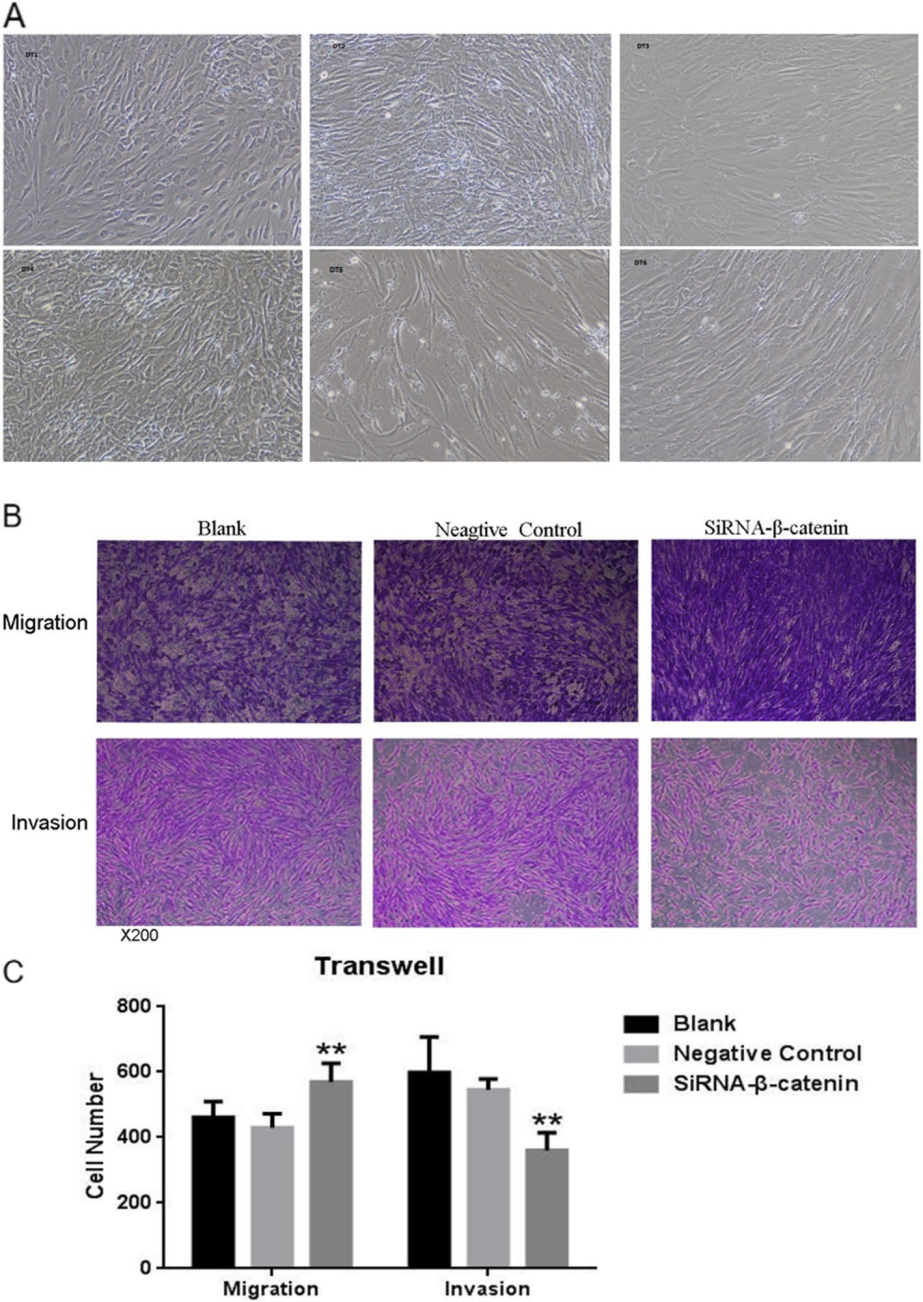
Establishment of the primary PDT cell lines and investigation of the function of CTNNB1. **A** The representative images of the established primary PDT cell lines. **B**-**C** The representative images and statistical analysis showed that silencing CTNNB1 significantly inhibits primary PDT cell invasion but promotes cell migration

### Establishment of the Primary PDT Cell Lines and Investigation of the Function of CTNNB1

There were few desmoid tumor cell lines available for further investigation, especially for PDTs. So we tried to establish the primary PDT cell lines. Finally, we successfully established six primary PDT cell lines, which have been continually passaged in many generations (Fig. 5A). All the established primary culture PDT cells were examined to ensure that most of the cells were representative of the primary PDTs by comparing them with the original tumor tissues with the CTNNB1 mutation. Next, we preliminarily investigated the function of CTNNB1 using shRNA targeting CTNNB1. Interestingly, we found that silencing CTNNB1 significantly inhibits primary PDT cell invasion but promotes cell migration, suggesting that Wnt/β-catenin signaling pathways are critical for the aggressive growth of PDTs (Fig. 7B and C). The function of different mutation of CTNNB1 in PDTs is investigating.

## Discussion

To better understand the molecular pathology of pediatric desmoid tumors, we applied integrated multi-omics analyses (WES, RNAseq and metabolomics) in our collected paired fresh-frozen DT T/MN samples. Besides the well-known CTNNB1, which was identified 100% mutation in our samples, we also identified several mutations, such as membrane-associated mucins (MUC4, MUC12), which may also play some role in the development and progression of pediatric desmoid tumors. A previous systematic analysis study of 204 cases of pediatric and adult desmoid type fibromatosis (DTF) also indicates that DTF may harbor a broader mutational spectrum beyond CTNNB1 mutations [17]. MUC4 has also been reported as a highly sensitive and specific marker for low-grade fibromyxoid sarcoma [18]. Besides Wnt/β-catenin signaling, the Notch pathway has also been reported as a potential therapeutic approach for desmoid tumors [19, 20].

Our RNAseq analysis paired pediatric desmoid tumors identified a panel of potential novel therapeutic targets, especially for the dysregulated FDA-approved drug targets, which are promising for testing the related drugs for the treatment of DTs. We are currently investigating the dysregulated genes using shRNA or CRISPR/Cas9 gene editing and further exploring the available drugs for potential targeting therapy. Previously, gene expression profiling analysis of adult DTs by cDNA microarrays and identified the correlation between gene signature with progression-free survival of patients. This molecular signature identified two groups with clearly distinct PFS in the two sets of subjects (PFS 2 years: 86% vs. 44%) [21]. We observed that there are around 46 Wnt/β-catenin signaling pathway-related genes significantly dysregulated in DTs. We also found that silencing CTNNB1 significantly inhibits DT cell invasion but promotes cell migration. However, another study showed that Wnt targets genes are not differentially expressed in DTs bearing different activating β-catenin mutations. Therefore, it is crucial to further decipher the mechanism of different CTNNB1 mutations contributing to DTs. CTNNB1 mutation has also been reported in other tumors like liver cancer [22]. A recent study shows that RUNX3 plays a pivotal role in the β-catenin S45F mutation caused apoptotic resistance [23]. We are currently using CRISPR/Cas9 mediated gene editing to target CTNNB1 mutagenesis, which will be useful for understanding the mechanisms of different CTNNB1 mutations underlying the development and progression of pediatric desmoid tumors [24].

The untargeted metabolomics profiling in matched DTs T/MN samples identified the key metabolites and pathways in pediatric desmoid tumors. Broad-spectrum metabolomics has been used to understand the differences among untreated cell lines from the desmoid tumor, normal fibroblasts, and unaffected fibroblasts. Treatment of these different cells with novel drugs Dasatinib and FAK Inhibitor 14 has been found to alter metabotype of all cell lines [25]. We have first compared the metabolomics difference of fresh frozen matched DTs T/MN samples from pediatric desmoid tumor patients. We identified a panel of differential metabolites and the potential metabolites biomarkers. Pathway enrichment analysis using Pathway-associated metabolite sets (SMPDB) also showed some critical pathways in DTs.

## Conclusion

This comprehensive study identified critical clinical prognostic factors for PDTs, and further integrated multi-omics studies identified potential novel prognostic biomarkers and therapeutic targets for PDTs.

## Supplementary Information

**Additional file 1.**

## Data Availability

The data supporting this study’s findings are available in the manuscript and the additional files or by request to corresponding authors.

## Abbreviations

DT: Desmoid tumor
DTF: desmoid-type fibromatosis
AF: aggressive fibromatosis
PDT: Pediatric Desmoid Tumor
TKIs: tyrosine kinase inhibitors
NSAIDs: non-steroidal anti-inflammatory drugs
WES: Whole Exome Sequencing
RNAseq: RNA sequencing
IPA: Ingenuity Pathway Analysis
