# Supplementary material for: Clinical Prognostic Factors and Integrated Multi-Omics Studies Identify Potential Novel Therapeutic Targets for Pediatric Desmoid Tumor
Source: Biol Proced Online. 2022 Dec 20;24:25. doi: 10.1186/s12575-022-00180-0 (PMC9768966; doi:10.1186/s12575-022-00180-0)
Supplement: Supplementary file 1 — Additional file 1. [file 12575_2022_180_MOESM1_ESM.docx]

**Clinical prognostic factors and integrated multi-omics studies identified potential novel therapeutic targets for pediatric desmoid tumor**

**Supplementary Materials and Methods**

**Pediatric desmoid tumor specimens and clinicopathological features**

We collected 98 Pediatric desmoid tumor patients who underwent complete resection at Children's Hospital of Fudan University (CHFU, Shanghai, China) from 2004 to 2020. This study was approved by the Institutional Review Board (IRB) at the CHFU. The sample collection was agreed with the guardian of each patient. All samples were confirmed with DT diagnosis by histopathology and molecular analysis based on the guidance. The clinicopathological features of 98 patients according to tumor presentations were shown in Table 1.

**Library preparation and Whole Exome Sequencing (WES)**

After isolation of genomics DNA, There are two main methods of QC for DNA samples. We test DNA degradation and potential contamination using Agarose Gel Electrophoresis and quantifies the DNA concentration precisely by Qubit 2.0 analysis. A total amount of 1.0μg genomic DNA per sample was used as input material for the DNA sample preparation. Sequencing libraries were generated using Agilent SureSelect Human All ExonV6 kit (Agilent Technologies, CA, USA) following manufacturer’s recommendations and x index codes were added to attribute sequences to each sample. Briefly, fragmentation was carried out by a hydrodynamic shearing system (Covaris, Massachusetts, USA) to generate 180-280bp fragments. The remaining overhangs were converted into blunt ends via exonuclease/polymerase activities and enzymes were removed. After adenylation of 3’ ends of DNA fragments, adapter oligonucleotides were ligated. DNA fragments with ligated adapter molecules on both ends were selectively enriched in a PCR reaction. Captured libraries were enriched in a PCR reaction to add index tags to prepare for hybridization. Products were purified using the AMPure XP system (Beckman Coulter, Beverly, USA) and quantified using the Agilent high sensitivity DNA assay on the Agilent Bioanalyzer 2100 system. The qualified libraries are fed into Illumina sequencers after pooling according to its effective concentration and expected data volume.

**Library preparation and RNA sequencing (RNAseq)**

After isolation of total RNA, there are three main methods of QC for RNA samples: (1) Nanodrop: Preliminary quantitation; (2) Agarose Gel Electrophoresis: tests RNA degradation and potential contamination; (3) Agilent 2100: checks RNA integrity and quantitation. After the QC procedures, mRNA from eukaryotic organisms is enriched using oligo(dT) beads. For prokaryotic organisms or eukaryotic organisms' long-non-coding libraries, rRNA is removed using the Ribo-Zero kit that cleaves the mRNA. First, the mRNA is fragmented randomly by adding fragmentation buffer, then the cDNA is synthesized by using mRNA template and random hexamers primer, after which a custom second-strand synthesis buffer (Illumina), dNTPs, RNase H, and DNA polymerase I are added to initiate the second-strand synthesis. Second, after a series of terminal repair, A ligation, and sequencing adaptor ligation, the double-stranded cDNA library is completed through size selection and PCR enrichment. The quality control of the library consists of three steps: (1) Qubit 2.0: tests the library concentration preliminarily. (2) Agilent 2100: tests the insert size. (3) Q-PCR: quantifies the library effective concentration precisely. The qualified libraries are fed into Illumina sequencers after pooling according to their effective concentration and expected data volume.

**The untargeted metabolomics profiling**

The untargeted metabolomics profiling was performed on the XploreMET platform (Metabo-Profile, Shanghai, China). The paired frozen pediatric desmoid tumor specimens were sent to the Metabo-Profile with liquid nitrogen, and the sample preparation procedures are referred in their protocols. All chemicals/reagents and more details are provided in the Supplementary Materials. The data analysis uses the XploreMET (v3.0, Metabo-Profile, Shanghai, China), which is a powerful 1-STOP solution for GC-MS-based metabolomics developed by the company. The raw data generated by GC-TOF/MS were processed using XploreMET for automated baseline denoising and smoothing, peak picking and deconvultion, creating reference database from the pooled QC samples, metabolite signal alignment, missing value correction and imputation, and QC correction. Each data set was transformed into comparable data vectors for statistical analysis. The software integrates one of the most extensive metabolite databases – JiaLib in the world and streamlines procedures for raw data processing, peak deconvulation, compound annotation, statistical analysis, pathway analysis, and project report within minutes of completing the analytical sequence.

**Establishment of the primary pediatric desmoid tumor cell lines**

Primary cell cultures from different DT tissue samples were collected during surgical resection. The DT tissues were immediately cut into small pieces and incubated with collagenase solution for 18 to 24 hours at 37 ^o^C. Then the cell suspension was filtrated through 100μm filters. The dissociated cells were divided into multiple culture dishes after the cells achieved near confluence and grown in DMEM with 15% FBS. The established primary culture cells were examined to ensure that most of the cells were representative of the primary pediatric desmoid tumor cells, by performing immunohistochemistry (IHC) staining and sequencing analysis.

**Transwell migration and invasion assay**

The control vector plasmid and CTNNB1 shRNA plasmids were obtained from Sigma. The established primary pediatric desmoid tumor cell lines were transfected with Lipofection 3000 and selected with 1 ug/mL puromycine (Life Technologies Inc.). The 24-well with 8.0-μm pore membrane transwell chambers was used according to the manufacturer's protocol (Corning USA). The 100μL of 1:8 DMEM-diluted Matrigel (BD, USA) needs to be added to each well for the transwell invasion assay. but no need to add for transwell migration assay. The stable transfected primary DT cells were seeded in the upper chamber in 100μL of serum-free medium, and 600μL of DMEM culture medium was added to the lower chamber as a chemoattractant at the same time. After incubated for 24 h at 37°C, the cells remaining at the upper surface of the membrane were removed with cotton swabs. The cells on the lower surface of the membrane are the migrated cells, which were fixed with 4% paraformaldehyde and stained with 0.1% crystal violet solution. The migrated cells were photographed by inverted fluorescence microscope and calculated.

**Bioinformatics and statistical analysis**

Raw sequencing output from WES and RNAseq was transferred from the sequencing instrument to a bioinformatics server for professional bioinformatics analysis. Briefly, WES data were aligned to the human reference genome build hg38 with bwa (version 0.7.15), followed by the GDC DNA-Seq analysis pipeline available from (https://docs.gdc.cancer.gov/). Somatic single nucleotide variants (SNVs) were identified using Varscan (version 2.3.9) and subjected to annotation via annovar. These SNVs were further filtered for missense and nonsense mutation and subjected to visualization via R (version 4.0.2) package maftools. The differential metabolites were obtained using univariate statistical analysis (student T-test or Mann-Whitney U test, depending on the normality of data and homogeneity of variance), especially when the multivariate OPLS-DA model fails to build a reliable discriminant model under some conditions. Pathway analysis for the significantly overexpressed genes was done by QIAGEN Ingenuity Pathway Analysis (QIAGEN IPA). Pathway enrichment analysis uses the Pathway-associated metabolite sets (SMPDB). MetaboAnalyst 5.0 Joint Pathway Analysis and Network Explorer were applied to performs integrated analysis on results obtained from combined metabolomics and gene expression studies[[1](#_ENREF_1)]. The statistical analysis softwares, Partek® Genomics Suite® and IBM SPSS Statistics, were used for further statistical analysis.
